# Strategies for the control of *Rhipicephalus microplus* ticks in a world of conventional acaricide and macrocyclic lactone resistance

**DOI:** 10.1007/s00436-017-5677-6

**Published:** 2017-11-20

**Authors:** Roger I. Rodriguez-Vivas, Nicholas N. Jonsson, Chandra Bhushan

**Affiliations:** 1 Facultad de Medicina Veterinaria y Zootecnia, Campus de Ciencias Biológicas y Agropecuarias, Universidad Autónoma de Yucatán, km. 15.5 Carretera Mérida-Xmatkuil, 97000 Mérida, Yucatán Mexico; 2 College of Medical, Veterinary and Life Sciences, University of Glasgow, G61 1QH, Glasgow, UK; 3 Bayer Animal Health GmbH, Kaiser-Wilhelm-Alee 10, 51368 Leverkusen, Germany

**Keywords:** *Rhipicephalus microplus*, Acaricides, Macrocyclic lactone, Resistance, Integrated tick management

## Abstract

Infestations with the cattle tick, *Rhipicephalus microplus*, constitute the most important ectoparasite problem for cattle production in tropical and subtropical regions worldwide, resulting in major economic losses. The control of *R. microplus* is mostly based on the use of conventional acaricides and macrocyclic lactones. However, the intensive use of such compounds has resulted in tick populations that exhibit resistance to all major acaricide chemical classes. Consequently, there is a need for the development of alternative approaches, possibly including the use of animal husbandry practices, synergized pesticides, rotation of acaricides, pesticide mixture formulations, manual removal of ticks, selection for host resistance, nutritional management, release of sterile male hybrids, environmental management, plant species that are unfavourable to ticks, pasture management, plant extracts, essential oils and vaccination. Integrated tick management consists of the systematic combination of at least two control technologies aiming to reduce selection pressure in favour of acaricide-resistant individuals, while maintaining adequate levels of animal production. The purpose of this paper is to present a current review on conventional acaricide and macrocyclic lactone resistance for better understanding and control of resistant ticks with particular emphasis on *R. microplus* on cattle.

## Introduction

Ticks are economically the most important pests of cattle and other domestic species worldwide (Jongejan and Uilenberg 1994). The FAO (1987) reported that more than 80% of the world’s cattle population is infested with ticks. The cattle tick *Rhipicephalus microplus* (formerly *Boophilus microplus*) is one of the most important livestock pests in tropical and subtropical areas of the world. Economic losses due to *R. microplus* are related to depression of milk production and liveweight gain, mortality, hide damage, morbidity, the cost of control and the effects of tick-transmitted haemoparasites (*Babesia bigemina*, *Babesia bovis* and *Anaplasma marginale*). Recently, in Brazil and Mexico, annual losses from tick infestation of *R. microplus* were estimated to be US$3.24 billion (Grisi et al. 2014) and US$573.61 million per annum (Rodriguez-Vivas et al. 2017), respectively.

Acaricides and macrocyclic lactones (MLs) have played an important role in the control of ticks. However, populations of several tick species mainly in tropical and subtropical countries have developed resistance to all major classes of these compounds due to the high intensity of their use in tick management (Rodriguez-Vivas et al. 2006a, b; Perez-Cogollo et al. 2010a). This has driven to the development of new chemical and non-chemical approaches to control. Integrated pest management involves the systematic application of two or more technologies to control tick populations which adversely affect the host species. The ultimate aim is to achieve parasite control in a more sustainable, environmentally compatible and cost-effective manner than is achievable with a single, stand-alone technology (Willadsen 2006). The purpose of this paper is to present an updated review on conventional acaricide and macrocyclic lactone resistance for better understanding and control of resistant tick species with particular emphasis on *R. microplus* on cattle.

## Chemical control of *Rhipicephalus microplus*

The chemicals used in the treatment of ectoparasites of veterinary importance act either systemically, following uptake of the compound from host tissues, or by direct contact with the target parasites following external application (Rodriguez-Vivas et al. 2014a). With the exception of acarine/insect growth regulators, virtually all ectoparasiticides are neurotoxins, exerting their effect on the ectoparasite nervous system (Taylor 2001). Traditional methods for the delivery of an acaricide treatment to cattle to control ticks required formulations such as a wettable powder, emulsifiable concentrate or flowable products. Currently used conventional acaricides and MLs can be applied to cattle by immersion of animals in a dipping vat, by hand-operated spray, in a spray race, by injection, as a pour-on, in an intraruminal bolus, as an ear tag, or using other pheromone–acaricide-impregnated devices (George et al. 2004). The major classes and general characteristics of conventional acaricides and MLs to control ticks on cattle are listed in Table 1.

**Table 1:** The major classes and general characteristics of conventional acaricides and MLs to control ticks on cattle worldwide

Drug classes	Active compounds	Characteristics
Organochlorines	(a) Chlorinated ethane derivatives: DDT, DDE (dichloro-diphenyldichloro-ethane) and DDD (dicofol, methoxychlor) (b) Cyclodienes, chlordane, aldrin, dieldrin, hepatochlor, endrin, toxaphene (c) Hexachlorocyclohexanes (HCH): benzene hexachloride (BHC) which includes the γ-isomer, lindane	A broad spectrum of activity on arthropods but are not free from toxicity; they are highly persistent in the environment, in milk and in meat, and may be retained in the fat of vertebrates (Beugnet and Franc 2012).
Synthetic pyrethroids	Type I. Lack an α-cyano group which is present at the phenylbenzyl alcohol position of type II pyrethroids (Soderlund et al. 2002). The main pyrethroid acaricides currently in use are the α-cyano-substituted pyrethroids such as cypermethrin, deltamethrin, cyhalothrin and flumethrin (George et al. 2004)	The spectrum of activity varies upon the molecules. Permethrin and deltamethrin are both insecticides and acaricides, whereas flumethrin is mainly an acaricide. Cypermethrin, deltamethrin and cyhalothrin are examples of SPs that are effective on susceptible ticks (> 98% efficacy) (Rodriguez-Vivas et al. (2014a). Flumethrin was designed for application to cattle as pour-on, but there is also an emulsifiable concentrate formulation that can be applied as a dip or spray. The active ingredient in the pour-on has a remarkable capacity for spreading rapidly on the skin and hair from points of application along the dorsal line of an animal to all areas of the body (George et al. 2004).
Organophospates	Ethion, chlorpyrifos, chlorfenvinphos and coumaphos are four of the most widely used OPs for treatment of tick-infested cattle (Abbas et al. 2014).	Can be extremely toxic in mammals. They are generally active against fly larvae, flies, lice, ticks and mites on domestic livestock and fleas and ticks on dogs and cats, although activity varies between compounds and differing formulations (MacDonald 1995).
Amidines	Among the formamidines, only amitraz is currently used for the control of cattle ticks (Jonsson and Hope 2007).	Amitraz is toxic against mites, lice and ticks in domestic livestock. It has been widely used on cattle in dips, sprays or pour-on formulations for the control of single-host and multi-host tick species (Taylor 2001). Amitraz continues to be one of the most popular acaricides for the control of *R. microplus* in Australia, southern Africa and Latin America (Jonsson and Hope 2007). Amitraz applied by aspersion to cattle infested with *R. microplus* had a therapeutic efficacy of 99.5–100% in the Mexican tropics (Aguilar-Tipacamu and Rodriguez-Vivas 2003).
Phenylpyrazoles	Fipronil is used worldwide for the treatment and control of flea and tick infestations on cattle, cats and dogs (Taylor 2001; George et al. 2004).	Fipronil applied as a pour-on to cattle infested with *R. microplus* had a therapeutic efficacy greater than 99% (Davey and George 1998).
Insect growth regulators (IGRs)	Based on their mode of action they are divided into (a) chitin synthesis inhibitors (benzoylphenyl ureas), (b) chitin inhibitors (triazine/pyrimidine derivatives) and (c) juvenile hormone analogues (Taylor 2001).	IGRs constitute a group of chemical compounds that do not kill the target parasite directly, but interfere with the growth and development. They act mainly on immature stages of the parasites and as such are not usually suitable for the rapid control of established adult populations of parasites. Fluazuron is efficacious against ticks and some mite species. The adverse consequences for ticks on cattle treated with a pour-on of this acaricide are the reduction of the fecundity and fertility of engorged females to near zero, and mortality of immature ticks because they unable to moult to the next instar (George et al. 2004).
Macrocyclic lactones	Avermectin: doramectin, selamectin, abamectin, ivermectin and eprinomectin Milbemycins: Moxidectin, milbemycin oxime Spinosyns: spinosad	MLs are broad-spectrum antiparasitic drugs widely used to control endoparasites and ectoparasites. The efficacy of ivermectin, doramectin and moxidectin for the control of *R. microplus* populations resistant to OPs, amidine and SPs has been demonstrated (Sibson 1994; Aguilar-Tipacamu and Rodriguez-Vivas 2003). In Mexico, moxidectin (1%) has been shown to have an efficacy against natural infestation of *R. microplus* greater than 95%, 28 days after application (Aguilar-Tipacamu and Rodriguez-Vivas 2003). Arieta-Román et al. (2010) showed that the long-acting moxidectin—10% (1 mg/kg) and ivermectin—3.15% (0.63 mg/kg) have an efficacy against natural infection of *R. microplus* greater than 95%, 70 and 56 days after applications, respectively. Eprinomectin is used against endo–ectoparasites without withdrawal time in milk and meat after its pour-on administration at 0.5 mg/kg (Davey and George 2002). In the USA, Davey et al. (2001) reported that spinosad applied topically to cattle using spray formulations proved effective to control cattle tick infestations.

Acaricide mixtures and synergized formulations have been also used to control ticks on cattle, although there is considerable variation among countries regarding the licensing and registration of mixtures. Simple modelling shows that the use of a hypothetical drug mixture, which might also have broader spectrum of activity, and against which there is no pre-existing detectable resistance, should extend the life of a formulation (McKenzie 1996). This theoretical argument does not carry much weight in practice; however, because in the present day, products are rarely formulated as mixtures until they have been on the market for some time. Consequently, the actual frequencies of resistance-conferring alleles are many orders of magnitude higher than those expected against a novel product and the actual benefit is unlikely to be perceptible. There is variation among countries in the extent to which regulatory standards allow for the registration of acaricide mixtures. Some of the mixtures that are commercially available include compounds with synergistic activity. Several organophosphates (OPs) synergize the toxicity to *R. microplus* of deltamethrin and cypermethrin. In Australia, a combination product containing deltamethrin, chlorfenvinphos, cypermethrin and ethion has been used to control *R. microplus* (George et al. 2004). In the USA, Davey et al. (2013) evaluated the efficacy of a mixture of OP acaricides (dichlorvos and tetrachlorvinphos) as a spray at 0.3 and 0.15% active ingredient on cattle infested with immature and mature parasitic stages of OP-resistant *R. microplus*. The overall percentage mortality provided by 0.3 and 0.15% of the active ingredient was 87.6 and 85.3%, respectively. Although this OP mixture provided useful control against a highly OP-resistant strain of ticks, the control fell short of the 99% level required for use in the US Cattle Fever Tick Eradication Program. In Brazil, the most common mixtures of synthetic pyrethroids (SPs) and OPs are formulations of cypermethrin and chlorpyriphos, with or without a synergist (i.e. pyperonylbutoxide (PBO)). In Brazil, a pour-on formulation of fluazuron + abamectin is available in the market (SINDAN 2013). In Mexico, mixtures of acaricides are available in the market and flumethrin + cyfluthrin, chlorpyriphos + permethrin and cypermethrin + cymiazole are the most used (Rodriguez-Vivas et al. 2006a).

## Acaricide resistance in *Rhipicephalus microplus*

### Definition of resistance

The definition of resistance has changed with time and remains the subject of discussion. In 1957, the WHO defined resistance as “the development of an ability to tolerate toxicants which would prove lethal to the majority of individuals in a normal population of the same species”. Later, in 1992, the WHO defined resistance in arthropods as “an inherited characteristic that imparts an increased tolerance to a pesticide, or group of pesticides, such that the resistant individuals survive a concentration of the compound(s) that would normally be lethal to the species”. In this paper, our definition of acaricide resistance is a specific heritable trait(s) in a population of ticks, selected as a result of the population’s contact with an acaricide, which results in a significant increase in the percentage of the population that survives after exposure to a given concentration of that acaricide. In a dose–response bioassay, it is considered that there is acaricide resistance when the 95% confidence limit of the 50% lethal dose of a tested population does not overlap that of a susceptible reference strain (Robertson et al. 2007). Nonetheless, reference will be made to other definitions (Rodriguez-Vivas et al. 2012a).

### Phenotypic and genotypic resistance

A distinction is made between the resistance phenotype and the resistance genotype. The resistance phenotype could be considered as how resistant or susceptible a tick is to the effects of an application of any given acaricide. The resistance genotype is the genetic composition of the tick, which leads to the expression of the resistance phenotype. It is important to note that the same resistance phenotype can be conferred by different genetic variants (Guerrero et al. 2014).

#### Phenotypic resistance

In bioassays, the evaluation of dose responses (mortalities) remains the most definitive method of quantifying acaricide resistance in a population of ticks drawn from the field and in which the frequencies of all possible resistance-conferring alleles are unknown. For routine diagnostics, molecular testing for specific mutations can only identify known mechanisms. Although each individual tick can be susceptible or resistant to a given dose of an acaricide, the resistance phenotype is usually quantified and expressed in terms of the phenotype of a tick population. There are two related ways of expressing this: (1) the proportion of ticks that are not killed by a given acaricide concentration (discriminating dose or DD) and (2) the ratio of the dose of acaricide required to kill a given proportion of a test population (i.e. 50, 90 or 99%) in comparison with a susceptible reference strain (Rodriguez-Vivas et al. 2012a; Guerrero et al. 2014).

**Fig. 1:**
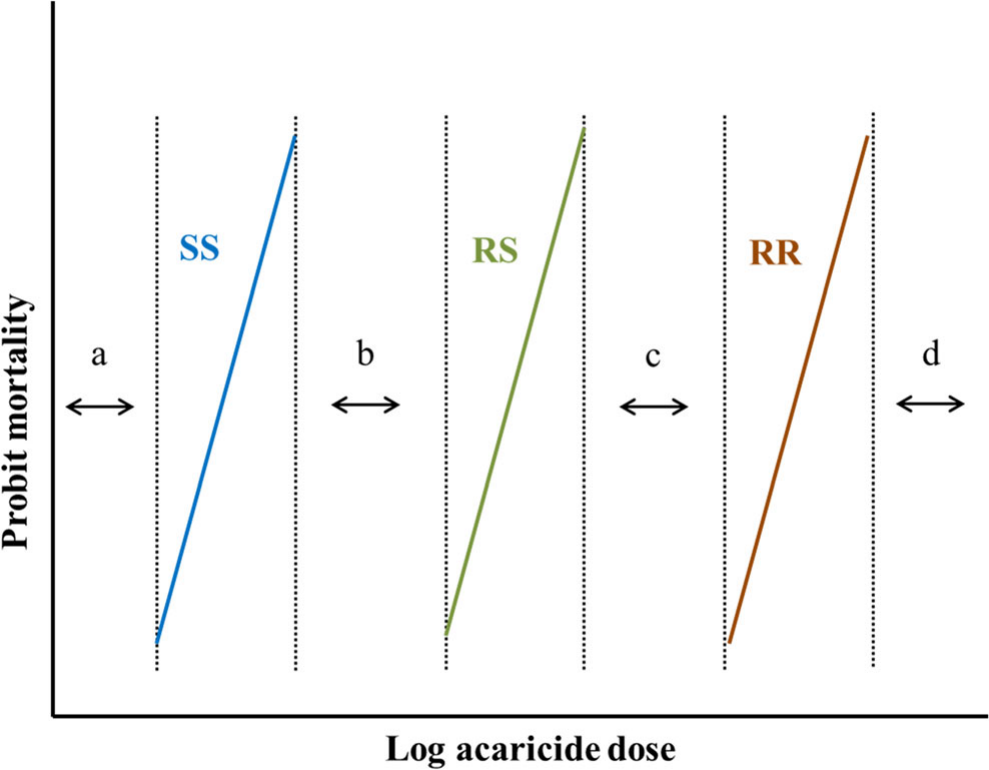
Four ranges of acaricide concentrations. **a** No mortality of any genotype (no selection). **b** Mortality of SS only (resistance dominant). **c** Mortality of RS and SS (resistance recessive). **d** All genotype killed (no selection)

In bioassays, there are four ranges of acaricide concentrations: (a) no mortality of any genotype (no selection), (b) mortality of SS and RS (resistance recessive), (c) mortality of SS only (resistance dominant) and (d) all genotypes killed (no selection) (Fig. 1).

The FAO (2004) recommended some specific bioassay techniques to test resistance to acaricides in ticks. The larval packet test (LPT) developed by Stone and Haydock (1962) has been used extensively for the diagnosis of resistance in field studies and also for the characterization of resistance mechanisms to SP and OP and in ticks. It is considered to be a highly repeatable bioassay technique (Jonsson et al. 2007), although it is limited by the labour and time required to obtain results (Guerrero et al. 2014). The larval immersion test (LIT) was developed by Shaw (1966) and is mainly used to characterize resistance mechanisms to macrocyclic lactones and amitraz (Rodriguez-Vivas et al. 2006a; Perez-Cogollo et al. 2010a). Recent modified LIT techniques using syringes have been developed to reduce the labour required for the traditional Shaw test (Sindhu et al. 2012). The use of microtiter plates has proven advantageous in automated high-throughput screening (White et al. 2004). Lovis et al. (2013) developed the larval tarsal test (LTT), a sensitive, efficient bioassay to enable high throughput of many compounds. The LTT produced resistance factors comparable to those obtained with the LPT. In the field, the adult immersion test (AIT) (FAO 2004) is probably the most widely used bioassay technique, although it has been shown to be a poor test (Jonsson et al. 2007). The AIT uses engorged female ticks which are immersed in technical or commercial acaricides (Guerrero et al. 2014).

The discriminating dose (DD) test uses any bioassay technique in which a single concentration, usually at double the LC_99.9_ or LC_99_ of a known susceptible strain is used to discriminate between susceptible and resistant tick populations (FAO 2004). The sample is either described as resistant or susceptible according to an arbitrary cut value, or as the percentage of larvae that survived the treatment (although this should not be taken to extend to the expected efficacy of the acaricide in the field). One major problem with this approach is the wide confidence intervals seen at LC_99.9_ for most bioassays. Hence, it is difficult (or impossible) to accurately determine a value for LC_99_ or LC_99.9_ with any confidence (Jonsson et al. 2007).

A full dose–response bioassay, in which replicates of ticks are exposed to serial dilutions of acaricide, is required to properly quantify the phenotypic resistance of *R. microplus* populations to acaricides and is an obvious prerequisite for the application of a discriminating dose method. Probit analysis is then used to determine the lethal concentration (LC) required to kill 50, 90 or 99% of the population (LC_50_, LC_90_ or LC_99_) (Robertson et al. 2007). The resistance ratio or resistance factor (RR or RF) is the “LC value of the tested sample divided by the LC value of a reference strain” (FAO 1987). Usually, the LC_50_ value is used for this purpose because it can be most accurately determined. The use of other LCs (i.e. LC_90_, LC_95_ or LC_99_) (Miller et al. 2007a; Cabrera-Jimenez et al. 2008; Rodriguez-Vivas et al. 2012b) and the slope (i.e. population response to increasing doses of the acaricide) (Robertson et al. 2007) are required to fully characterize the resistance.

Various arbitrary criteria have been proposed to evaluate the resistance level of *R. microplus* to acaricides. Beugnet and Chardonnet (1995) considered tick populations to be susceptible to SP when RF values (measured at the LC_50_) were < 3.0, tolerant 3–5 and resistant ≥ 5.0. For SP, Rodriguez-Vivas et al. (2012b) recommended using RFs for both LCs (LC_50_ + LC_99_). They considered populations to be susceptible when both RF values (judged by LC_50_ and LC_99_) were < 3.0 and resistant when RF values were > 5.0. Populations were considered tolerant when one or both RF values were 3–5. Castro-Janer et al. (2011) suggested using the following criteria for ivermectin resistance: susceptible RF_50_ ≤ 1, low resistance RF_50_ > 1 ≤ 2 and resistant RF_50_ > 2. Resistance ratios for SPs are high compared with compared with OP, amitraz and MLs, and substantial inter-population variation in the phenotypic level of acaricide resistance has been reported worldwide (Table 2).

**Table 2:** Phenotypic level of acaricide resistance (resistance factor) in *R. microplus* reported worldwide

Ixodicides or MLs	RF_50_	RF_90_	RF_99_	Author	Country
Phenylpyrazoles					
Fipronil	4.6	–	8.5	Miller et al. (2013)	USA
	0.7–1.5	0.8–2.0	–	Lovis et al. (2013)	Argentina
	1.8	–	0.9	Rodriguez-Vivas et al. (2013)	Mexico
Pyriprol	0.7–2.5	0.5–1.9	–	Lovis et al. (2013)	Argentina
Pyrethroids					
Cypermethrin	0.3–2599	–	0.7–5000	Rodriguez-Vivas et al. (2012b)	Mexico
	> 246	–	> 72.2	Rodriguez-Vivas et al. (2013)	Mexico
	1.7–57.0	2.1–116.2	–	Lovis et al. (2013)	Argentina
	8.7–33.9	38.3–48.8	–	Lovis et al. (2013)	Australia
Flumethrin	0.9–23.0	0.2–46.3	–	Lovis et al. (2013)	Argentina
	23.0–43.4	51.5–58.3	–	Lovis et al. (2013)	Australia
Deltamethrin	8.3–97.7	–		Beugnet and Chardonnet (1995)	New Caledonia
Permethrin	–	9.5*	–	Miller et al. (2007b)	USA
Macrocyclic lactones					
Ivermectin	7.0–10.2	–	50.2–179.6	Perez-Cogollo et al. (2010a)	Mexico
	2.6–3.0	–	9.5–6.5	Fernandez-Salas et al. (2012a)	Mexico
	7.1.	–	5.0	Rodriguez-Vivas et al. (2013)	Mexico
	1.8–4.6	–	–	Klafke et al. (2011)	Brazil
	1.3–1.9	–	–	Castro-Janer et al. (2011)	Uruguay
Organophosphates					
Coumaphos	2.8–10.0			Li et al. (2003)	Mexico
	3.6	5.0	6.5	Miller et al. (2005)	USA
	6.8	–	5.9	Rodriguez-Vivas et al. (2013)	Mexico
	5.6–6.4	7.5–16.0	–	Lovis et al. (2013)	Australia
Diazinon	6.3–34.4			Li et al. (2003)	Mexico
	7.1	11.7	17.7	Miller et al. (2005)	USA
	1.3–5.4	1.0–4.3	–	Lovis et al. (2013)	Argentina
Chlorphyriphos	1.5		0.6	Rodriguez-Vivas et al. (2013)	Mexico
Amidines					
Amitraz	1.0–4.5	–	–	Li et al. (2004)	USA
			41.9	Soberanes et al. (2002)	Mexico
	1.0–22.0	–	–	Rosado-Aguilar et al. (2008)	Mexico
	2.3	–	4.4	Rodriguez-Vivas et al. (2013)	Mexico
	0.7–32.5	–	0.1–4.3	Lovis et al. (2013)	Argentina

*RF*_*50*_ resistance factor at 50%, *RF*_*90*_ resistance factor at 90%, *RF*_*99*_ resistance factor at 99%, − no available data, *USA* United States of America

*In the F_2_

#### Genotypic resistance

Increasingly, it is possible to describe the genotypic resistance profile of a tick or a population of ticks as molecular markers for resistance status become available. The first markers of resistance were developed for SPs. He et al. (1999) studied the molecular mechanism of resistance to SPs in *R. microplus* and obtained and sequenced a partial *para*-homologous sodium channel cDNA from susceptible and SP-resistant strains. A point mutation (T2134A) that results in an amino acid change (F → I) was identified in a highly conserved domain III segment 6 of the homologous sodium channel gene from ticks that were resistant to SPs (He et al. 1999). This was followed by the discovery of two new SNPs in domain II segments 4 and 5 (C190A) of the linker region of the sodium channel gene in *R. microplus* (Morgan et al. 2009; Jonsson et al. 2010a). Stone et al. (2014) studied *R. microplus* populations from the USA and Mexico and found resistance-conferring SNPs in domains II and III of the *para*-sodium channel gene associated with SP resistance. Additionally, the authors discovered a putative *super-kdr* SNP in domain II (T170C). Recently, van Wyk et al. (2016) found that the C190A mutation within domain II of the sodium channel is the main pyrethroid resistance mechanism for *R. microplus* in South African tick populations.

Molecular genetic markers for OP resistance have been slower to emerge, reflecting a higher degree of complexity of the OP–target–detoxification system. Point mutations in the gene encoding acetylcholinesterase (AChE) that result in production of an altered enzyme have been shown to be a major mechanism of OP resistance in several insects (Temeyer et al. 2007). Baxter and Barker (1998) isolated the first putative AChE gene (AChE1) in *R. microplus* larvae from Australia. This was the first report of alternative splicing in an AChE gene from *R. microplus*. Two other putative *R. microplus* AChE genes (AChE2 and AChE3) have since been discovered (Hernandez et al. 1999; Temeyer et al. 2004). Temeyer et al. (2010) expressed three acetylcholinesterase-like transcripts isolated from two OP-resistant and one OP-susceptible strain of *R. microplus* and showed that variant alleles existed among individuals in a strain that showed differential response to OP. The availability of the cDNA sequences for susceptible or OP-insensitive AChEs allowed rapid identification of OP resistance mutations in AChEs responsible for OP insensitivity and development of rapid molecular assays to determine the presence of specific OP-resistant mutations. Four (HQ184947, HQ184946, HQ184944, HQ184943) novel amino acid substitutions were identified in the AChE2 gene of resistant field isolates collected from the state of Bihar, India (Ghosh et al. 2015). Recently, Singh et al. (2016) reported six point mutations in the gene AChE3 in strains of *R. microplus* from India (I48L, I54V, R86Q, V71A, I77M and S79P), in which the first three were previously associated to resistance against OPs in the Mexican San Roman strain (Temeyer et al. 2007) and the other three were reported for the first time. Nagar et al. (2016) studied the role of mutations in esterase genes (carboxylesterase and AChE2) in the development of OP resistance in *R. microplus* ticks from India. Four amino acid substitutions (viz. V297I, S364T, H412Y and R468K) were found in AChE2 gene of resistant field isolates and in reference resistant lines.

There are four potential mechanisms of resistance to amitraz: (1) octopamine/tyramine receptor insensitivity, (2) beta-adrenergic octopamine receptor (*BAOR*) insensitivity, (3) elevated monoamine oxidase expression and (4) increased activity of ATP-binding cassette transporters (Jonsson et al. 2018). Baxter and Barker (1999) sequenced a putative octopamine receptor from amitraz resistant and susceptible *R*. *microplus* Australian strains and found no differences. However, as noted by Corley et al. (2012), the gene that was sequenced was more likely an octopamine-tyramine receptor. Chen et al. (2007) reported mutations in amitraz-resistant *R. microplus* in the same octopamine-tyramine receptor as examined by Baxter and Barker (1999). Corley et al. (2013) subsequently sequenced the *BAOR* gene and discovered a mutation in the first extracellular domain of the receptor that was predicted to result in an I61F substitution in amitraz-resistant *R. microplus*. Recently, Baron et al. (2015) confirmed that the two SNPs in octopamine-tyramine receptor reported by Chen et al. (2007) were associated with amitraz resistance in the South African tick strain. Recently, Robbertse et al. (2016) evaluated the acaricide resistance status and the level of genetic diversity in a partially isolated *R. microplus* population in 12 dip stations in South Africa. Approximately half of the ticks sampled proved to be genotypically resistant to amitraz on the basis of the presence of the SNPs described by Chen et al. (2007). Jonsson et al. (2018) describe a group of mutations in the *BAOR* in the same region as the first detected mutation, all associated with elevated resistance to amitraz. At present, polymorphisms in octopamine-tyramine receptor and *BAOR* have some potential for molecular diagnosis of amitraz resistance; however, the diversity of mutations suggests that no single polymorphism can be relied on.

In arthropods, γ-aminobutyric acid (GABA) is an inhibitory neurotransmitter at neuromuscular junctions and synapses in the central nervous system. Fipronil, dieldrin and isoxazoline chemical class (fluralaner) are reported to be antagonists of GABA-gated chloride channels in *R. microplus* (Ozoe et al. 2010). Mutations of the GABA gene of *Drosophila melanogaster* and *Anopheles funestus* have been reported (Wondji et al. 2011). Hope et al. (2010) reported mutations associated with dieldrin resistance in *R. microplus*. A mutation in the GABA-gated chloride channel gene was identified at position 868-9 and causes a Thr → Leu amino acid substitution.

The genotypic basis of resistance to MLs in arthropods has not been clarified (Rodriguez-Vivas et al. 2014a). Insensitivity of the GluCl receptor, which prevents drug binding to its target site, has been associated with ivermectin resistance in some nematodes and arthropods (Kwon et al. 2010). It has been suggested from molecular, pharmacokinetic, and biochemical studies that the most important molecules involved in detoxification of MLs are ATP-binding cassette (ABC) transporter proteins (Dermauw and Van Leeuwen 2014). The ABC transporter efflux pump is a defense mechanism against ivermectin in *R. microplus* (Pohl et al. 2012), and variation in the level of expression of the *ABCB10* gene has been associated with resistance to MLs in ticks (Pohl et al. 2012) and to other acaricides using in vitro approaches in cell cultures (Koh-Tan et al. 2016)*.* However, despite the evidence of altered *ABCB10* expression in resistant populations, the genotypic genotypic basis of this variation is not known, and there are no useful molecular diagnostic tests for resistance to MLs.

#### Correlation between genotypic and phenotypic resistance

Strong correlations between the frequency of resistance-conferring alleles in samples of ticks and their resistance phenotype in a bioassay (have been reported for the *para*-sodium channel gene, for the octopamine gene and for the *BAOR*). In Mexico, Rosario-Cruz et al. (2005) working with nine populations of *R. microplus* found a positive correlation (flumethrin *r* ^2^ = 0.849; cypermethrin *r* ^2^ = 0.856; deltamethrin *r* ^2^ = 0.887) between larval survival (using DD) and the percentage of the resistant allele of the sodium channel mutation known to be involved in SP resistance. Li et al. (2007) found a significant correlation (*r* ^2^ = 0.827) between the permethrin resistance factor and allele frequency of the T2134A mutation in five laboratory strains of *R. microplus*. In a study carried out in Mexico, Rosario-Cruz et al. (2009) found that the presence of the T2134A mutation of *R. microplus* was associated with resistance to flumethrin, deltamethrin and cypermethrin. Rodriguez-Vivas et al. (2012b) studied the prevalence of pyrethroid resistance phenotype and genotype in *R. microplus* in Yucatan, Mexico, and found that the increasing presence of the resistance allele correlated well with increased levels of dose response to cypermethrin. Rodriguez-Vivas et al. (2011) studied the phenotypic and genotypic changes in field populations of *R. microplus* in response to SP selection pressure. The authors found a strong correlation between the percentage of homozygous resistant ticks and the proportion of larval survival in three of four studied tick populations (*r* ^2^s = > 0.850), confirming that the T2134A mutation is a major cause of SP resistance in Mexico. In Australia, Morgan et al. (2009) and Jonsson et al. (2010a) studied field populations of *R. microplus* with synthetic pyrethroid resistance status and found close correlations between the *para*-sodium channel gene mutations and survivorship in larval bioassays.

In Queensland, Australia, Corley et al. (2013) found a positive correlation between the frequency of the I61F-resistant homozygous genotype in the beta-adrenergic-like octopamine receptor and resistance of *R. microplus* to amitraz (*r* = 0.90).

#### Cross-resistance and multiple resistance

Cross-resistance is when the exposure of a population to one compound leads to the selection of adaptations that confer resistance to a different compound. Multiple resistance occurs when ticks develop resistance to two or more than two compounds by expressing multiple resistance mechanisms. Multiple resistances of different classes of acaricidels used to control ticks have become increasingly prevalent worldwide. Table 3 lists reports of cross-resistance and multiple resistance in *R. microplus* to acaricide and ML in different parts of the word.

**Table 3:** Cross and multiple resistance of *R. microplus* to conventional acaricide and ML reported worldwide

Field population or laboratory strain (number)	Acaricide or ML (test used to diagnose resistance)	Country	Reference
Ultimo strain	SP (LPT) + AM (LPT)	Australia	Kunz and Kemp (1994)
Coatzacoalco strain	OP (LPT) + SP (LPT)	USA	Miller et al. (1999)
Mora strain	OP (LPT) + SP (LPT)	Mexico	Redondo et al. (1999)
Montecitos strain	OP (LPT) + SP (LPT) + AM (AIT)	Colombia	Benavides et al. (2000)
Field populations	AM (LIT) + OP (LPT) + SP (LPT)	Mexico	Rodriguez-Vivas et al. (2007)
Field populations	IVM (LIT) + PYZ (LIT)	Uruguay	Castro-Janer et al. (2011)
Field populations	OP (LPT) + SP (LPT)	Brazil	Mendes et al. (2011)
Field populations	OP (LPT) + SP (LPT) + AM (AIT) + IVM (LIT)	Mexico	Fernandez-Salas et al. (2012b)
Field populations	SP (AIT) + AM (AIT)	Brazil	Veiga et al. (2012)
Field population	OP (LPT) + SP (LPT) + AM (LIT) + IVM (LIT) + PYZ (LPT)	Mexico	Rodriguez-Vivas et al. (2013)
Santo Tomé strain	SP (AIT, LTT) + AM (AIT, LTT)	Argentina	Cutullé et al. (2013)
Field populations	SP (LTT) + PYZ (LTT)	South Africa	Lovis et al. (2013)
Field populations	OP (LTT) + SP (LTT)	Australia	Lovis et al. (2013)
Field populations	OP (LPT) + SP (LPT) + AM (LPT) + IVM (LI) + PYZ (LPT) + Fluazuron (AIT)	Brazil	Reck et al. (2014)
Zamora strain	OP (LPT, EST) + SP (LPT) + AM (LPT) + PYZ (LPT)	Mexico	Miller et al. (2013)
Filed populations	OP + SP (LPT), SP + AM + PYZ (LPT), OF + SP + PYZ (LPT)	USA	Busch et al. (2014)
Field population	OP (LPT) + SP (LPT) + AM (LIT) + IVM (LIT)	Mexico	Fernandez-Salas et al. (2012b)

*ML* macrocyclic lactone, *OF* organophosphates, *SP* synthetic pyrethroids, *AM* amidine, *IVM* ivermectin, *PYZ* phenylpyrazoles, *EST* esterase, *LPT* larval packet test, *AIT* adult immersion test, *LIT* larval immersion test, *LTT* larval tarsal test

### Factors influencing the rate of emergence of resistance to acaricides

The rate at which a resistant allele becomes established in the population and the time it takes for the control of ticks to break down is dependent upon (a) the frequency of the original mutation in the population before treatment, (b) the mode of inheritance of the resistant allele, (c) the proportion of the total tick population that is exposed to the acaricide, (d) the frequency of acaricide treatment and (e) the rate of dispersal of resistant ticks into new areas. Emergence of resistance to acaricides can be seen as an evolutionary process, subject to the main drivers of population genetics: (1) mutation, (2) drift, (3) selection and (4) migration. Of these factors, mutation relates to the initial frequency of resistance-conferring alleles; selection is a function of the mode of inheritance, refugia, frequency and concentration; migration is dispersal. Drift (loss of rare alleles and fixation of common alleles at a locus) has not been investigated to any great extent in tick populations, but is likely to be particularly relevant to the genetics of tick strains maintained in culture and the genetics of outbreak populations in previously uninfested areas.

#### Initial frequency of resistance-conferring alleles

The initial frequency of resistance-conferring alleles in a population is one of the most important determinants of the rate of emergence of resistance when selection is applied (Roush and McKenzie 1987). It is expected that alleles that will confer resistance to any compound are already present at very low levels in the tick population before the introduction of a new acaricide. Estimates of initial frequencies of resistance-conferring alleles in naïve populations of arthropods range considerably, from 10^−2^ to 10^−13^ (Roush and McKenzie 1987; Gould et al. 1997). To confirm an initial frequency of 10^−3^ would require something between 1000 and 10,000 tests, which explains why empirical data from the field are scarce. Gould et al. (1997) used 2000 single-pair matings and a bioassay to detect alleles conferring resistance to BT toxin in *Heliothis virescens*, resulting in a high estimate of initial frequency of 1.5 × 10^−3^. This high frequency was proposed to have arisen from prior exposure of the population to related compounds. No initial frequencies of resistance-conferring alleles for any acaricide compounds have been determined for *R. microplus*.

#### Mode of inheritance

The mode of inheritance of resistance in *R. microplus* is the subject of several relevant studies. An acaricide resistance phenotype may be inherited as a dominant, partially dominant or recessive character (ffrench-Constant and Roush 1990). However, these classifications are more complex than is initially apparent. This is nicely illustrated in a figure taken from Roush and McKenzie (1987) that shows the effect of bioassay concentration on the apparent mode of inheritance of resistance for a monogenic resistance mechanism (Fig. 1). In the field, things are messier than they are in the laboratory and the concentrations to which ticks are exposed vary widely. Hence, the mode of inheritance determined from laboratory bioassays may not reflect the mode of inheritance actually seen under field conditions. The mode of inheritance of SP compounds in the field has been reasonably well described. Early work (e.g. Tapia-Perez et al. 2003) suggested that resistance was polygenic, but more recent work (e.g. Rodriguez-Vivas et al. 2012b) has confirmed that most cases of resistance in the field can be attributed to one of four known allelic variants of the *para*-sodium channel gene (He et al. 1999; Morgan et al. 2009; Jonsson et al. 2010a; Stone et al. 2014). Based on reciprocal crosses of a susceptible and a resistant *R*. *microplus* strain, Aguilar-Tipacamu et al. (2008) evaluated the inheritance of SP resistance using the ‘effective dominance of survival method’ described by Bourguet et al. (2000). The authors found that pyrethroid resistance (cypermethrin, flumethrin and deltamethrin) is inherited as a partially dominant trait when the *R. microplus* female is resistant. However, when the male is resistant for flumethrin and deltamethrin, the resistance is inherited as complete recessive (partially dominant for cypermethrin). The molecular studies of Morgan et al. (2009) and Jonsson et al. (2010a) strongly suggest a recessive mode of inheritance for the phenotypes arising from these mutations, at least in standard bioassays of SP efficacy. Li et al. (2004, 2005) suggested that amitraz resistance was inherited as an incomplete recessive trait; however, Fragoso-Sanchez et al. (2011) found that amitraz resistance in *R. microplus* is almost completely recessive; the work of Corley et al. (2013) with *BAOR* also indicated a recessive mode of inheritance for amitraz resistance.

#### Selection intensity—field and laboratory studies

Selection intensity for acaricide resistance is driven strongly by the frequency of acaricide applications and by the proportion of ticks that are untreated at any time when treatments are applied (Kunz and Kemp 1994). The proportion of ticks that are not exposed to any acaricide treatments is known as the refugia. Whereas many studies have been applied in the laboratory, relatively few have been conducted in the field. The following paragraphs briefly describe some studies on the application of selection pressure with the main classes of acaricide to *R. microplus.*

##### Organophosphates

Under laboratory conditions, Harris et al. (1988) conducted a study to generate resistance in *R. microplus* to OPs. The authors selected for resistance to coumaphos by dipping groups of engorged *R. microplus* females in serial dilutions (0.2, 0.1, 0.06, 0.03 and 0.01% of active ingredient) prepared from a commercial 50% flowable formulation of coumaphos. Surviving offspring from females treated with the most concentrated coumaphos dilutions were retained for reproduction. This method of selection was used for the three generations in the laboratory; then, the authors changed to a technique in which larvae from a single female were selected and treated with coumaphos (0.1 to 1%). During 12 generations with selection process, the studied strain of *R. microplus* became 38 times more resistant to coumaphos than the susceptible reference strain. Working with a resistant strain (‘Tuxpan’), Wright and Ahrens (1989) made selection pressure in three generations by dipping groups of engorged females in dilutions of 42% (active ingredient) flowable formulation of coumaphos. They found that Tuxpan strain became more resistant to coumaphos as the generations proceeded. In another study conducted by Davey et al. (2003), larvae from F_1_ generation and all subsequent generations up to the F_14_ generation were selectively exposed to coumaphos (0.2 to 0.45%) to maintain or increase the amount of OP resistance in the strain. The F_2_ resulted in an estimated LC_50_ of 0.623%, whereas ticks in the F_14_ generation resulted in an estimated LC_50_ of 0.688%. Comparison of these results with the OP-susceptible reference strain revealed that the F_2_ generation of OP-resistant ticks was approximately 12 times more resistant to coumaphos than the OP-susceptible strain, whereas the F_14_ generation was approximately 13 times more resistant to coumaphos than the susceptible strain. Therefore, although the 12 successive generations of continuous selective exposure to coumaphos maintained the RF, it did not substantially increase the RF. Davey et al. (2004) worked with the same OP-resistant strain and applying pressure with coumaphos treatments during all 22 subsequent generations and found that the level of resistance did not significantly increase.

##### Amitraz

In laboratory conditions, Li et al. (2004) applied selection pressure using amitraz on larvae of a *R. microplus* strain (‘Santa Luiza’). The strain was challenged with different concentrations of amitraz and responded to selection quickly. The RF increased from 13.3 in F_1_ to 154 in F_6_. Although resistance decreased sharply without selection in the following generations (F_8_ = 68.72) and at low dose pressure of amitraz (F_9_ = 50.7, F_12_ = 49.43). In the Mexican tropics, Rosado-Aguilar et al. (2008) treated three field populations of *R. microplus* with amitraz. After 15 months of amitraz selection pressure, the three populations increased their RFs (from 1 to 13, from 1 to 22 and from 2 to 6). Fragoso-Sanchez et al. (2011) described the genetics of amitraz resistance evolution in *R. microplus*. They studied three Mexican tick strains, one susceptible to all acaricides and two amitraz resistant. Larvae were reared on isolated heifers and maintained nine generations in laboratory conditions. From each generation and each strain, the amitraz LC_50_ was chosen as the selection concentration for each strain. After 10 generations, the RFs increased 1–10, 4–60 and 10–107 for the susceptible and resistant (Palenque strain) and resistant (San Alfonso strain), respectively. In Queensland, Australia, Corley et al. (2013) found an increase over time in the frequency of the resistant homozygous I61F genotype in farms on which amitraz was used regularly, contrasted with relatively static frequency of the I61F homozygous genotype in farms on which amitraz was never used. In this study, the authors showed a strong association between a polymorphism in a highly conserved region of the RmβAOR gene of *R. microplus* and resistance to amitraz in the larval packed test and demonstrated that the mutation is selected for by treatment with amitraz over seven generations in the field.

##### Synthetic pyrethroids

In a controlled field trial, Coetzee et al. (1987) reported rapid onset and development of fenvalerate in *B. decoloratus.* The selection for resistance occurred during an 18-month period (equivalent to five to six generations). Davey and George (1998) selected a *R. microplus* strain for resistance to permethrin by treating larvae with increasing doses (range, 0.05–0.35%) through successive generations (generations F_2_–F_7_). At the beginning of the selection process (F_2_), the SP-resistant strain was 5.4 times more resistant to permethrin than the SP-susceptible strain, and the level of resistance increased in each successive generation of the SP-resistant strain, reaching a RF of 20.9 in the F_7_ generation. In a prospective controlled intervention field study, Rodriguez-Vivas et al. (2011) measured the resistance phenotype and genotype of *R. microplus* on 11 farms in Yucatan, Mexico, where cypermethrin was used regularly. On five farms, cypermethrin continued to be used, and on six, it was substituted with amitraz used every 30–45 days. After 24 months of continued selection pressure with cypermethrin, the RF increased from 2-fold to 125-fold. The frequency of the resistance-conferring allele (T2134A mutation) increased on all five farms from a starting range of 6–47% to a range of 66–95% after 24 months. On six farms treated with amitraz, neither the SP RFs nor the frequency of the T2134A allele changed significantly. It was concluded that SP selection pressure on a field population of *R. microplus* rapidly generated cypermethrin resistance with increases of RF which correlated with increased frequencies of the resistance allele. In populations in which cypermethrin was substituted, other acaricide class (amitraz) RFs and frequencies of the resistance allele remained stable over 24 months.

##### Macrocyclic lactones

At present, the only study reporting selection intensity for ivermectin resistance was conducted in Brazil by Klafke et al. (2010). The authors used four methodologies to select the ivermectin-resistant strain: (1) cattle infestation with IVM-treated larvae, (2) with larvae from IVM-treated adult female ticks, (3) with larvae from IVM-treated adult female ticks on an IVM-treated host and (4) with larvae obtained from IVM-treated females that produced eggs with a high eclosion rate. After ten generations of *R. microplus*, using these methods combined the RF increased from 1.37 to 8.06.

#### Risk factors for acaricide resistance derived from field studies

Jonsson et al. (2000) and Bianchi et al. (2003) identified several factors associated with increased probability of resistance to different acaricides. The risk factors differed among the acaricides tested, frequency of application, type of application, farm localization, fly control and grazing management. Rodriguez-Vivas et al. (2006a) found in the Mexican tropics high probability of *R. microplus* SP resistance on farms where acaricides were applied ≥ 6 times in 1 year (OR = 4.83). This finding is in agreement with Sutherst (1979), which indicated stronger selection for resistance when six acaricide applications were made per year, compared with four or five applications per year. Similar results were found by Jonsson et al. (2000) who found higher probability of tick resistance to cypermethrin, deltamethrin and flumethrin when acaricides were used > 5 times/year. However, it was noted that the first response of many farmers to a problem of acaricide resistance is to increase the frequency of treatment, making it difficult to distinguish between cause and effect in observational, cross-sectional studies. Fernandez-Salas et al. (2012a) found that on cattle farms of Veracruz, Mexico, those which used ML ≥ 4 times per year were more likely to develop *R. microplus* resistant to ivermectin (OR = 13.0). Rodriguez-Vivas et al. (2006a) also found in farms that used another tick control program were associated with higher probability of *R. microplus* presenting flumethrin, deltamethrin and cypermethrin resistance (OR = 5.9).

#### Persistence of insecticide resistance

Whereas selection pressure with an acaricide is expected to increase the frequency of resistant genotypes in a population, it is possible that removal of the selection pressure might be followed by a reduction in the frequency of the resistant genotypes, particularly if these genotypes are otherwise of lower reproductive fitness than the acaricide-susceptible genotypes in the absence of selection. Fitness costs associated with pesticide resistance have been documented in many pest species (Coustau et al. 2000; Oliveira et al. 2007). The reproductive fitness of *R*. *microplus* strains resistant to OPs, SPs or amitraz was compared to an acaricide-susceptible strain to determine whether the acquisition of resistance affected reproductive fitness in the resistant strains (Davey et al. 2006). The authors found that the OP-resistant strain produced 30% fewer eggs than the susceptible strain indicating that the acquisition of resistance placed the OP resistant at a selective disadvantage relative to the susceptible strain. The fitness cost of SP and amitraz-resistant strains was not found. However, Soberanes et al. (2002) reported in Mexico that the level of resistance of *R. microplus* to amitraz in the San Alfonso strain decreased from 42-fold to 10-fold after six generations on laboratory condition without amitraz selection. In field populations of *R*. *microplus*, Rodriguez-Vivas et al. (2005) found persistent resistance to OP for more than 4 years. Rodriguez-Vivas et al. (2011) used a tactical management strategy to reduce the cypermethrin resistance on field populations of *R*. *microplus* in the Mexican tropics. Cattle with pyrethroid-susceptible ticks were introduced into two farms with pyrethroid-resistant population over 31 months. This management caused significant reduction in RFs in farm 1 (LC_50_ = from 14.2 to 1.3) and farm 2 (LC_50_ = from 12.3 to 1.6). In farm 1 and farm 2, the frequency of the R allele (T2134A mutation) decreased from 56.7 to 15.5% and from 57.8 to 18.3%, respectively. In Queensland, Australia, Corley et al. (2013) studied the evolution of resistance to amitraz in *R. microplus* in field condition and tested the association between amitraz resistance and the frequency of the I61F mutation. Over the 3-year field study, there was some evidence of loss of resistance to amitraz in populations of ticks on farms where cattle were treated with spinosad.

## International reports of acaricide resistance

**Fig. 2:**
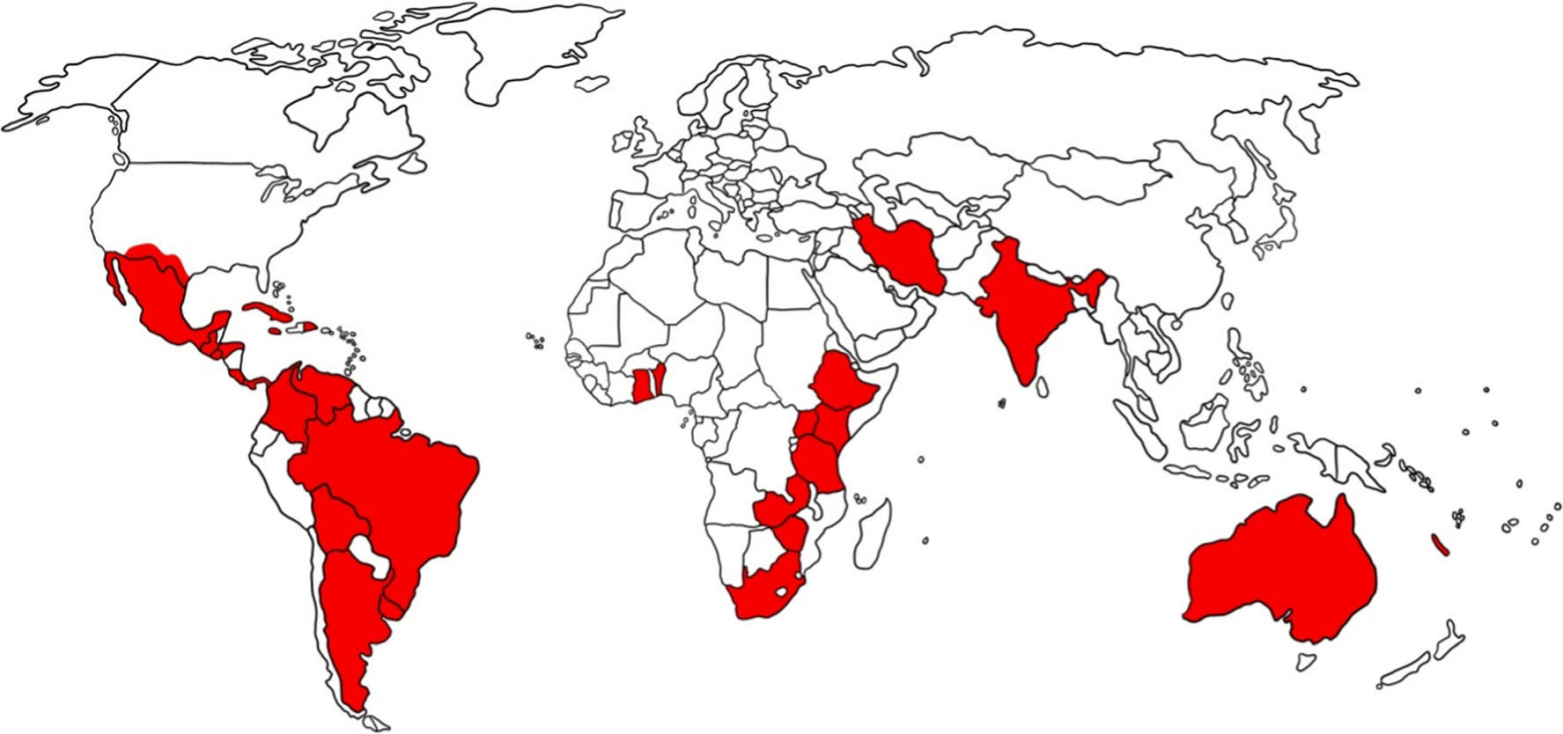
Geographic distribution of acaricide resistance in *Rhipicephalus* ticks worldwide (*R. microplus*: the USA, Mexico, Jamaica, Republica Dominicana, Cuba, Guatemala, Honduras, El Salvador, Panama, Costa Rica, Colombia, Venezuela, Bolivia, Uruguay, Brazil, Argentina, Australia, New Caledonia, India, Ira, Benin, Tanzania, South Africa and Zambia; *R. bursa*: India and Iran; *B. decoloratus*: Ethiopia, Zimbabwe, Tanzania, South Africa and Zambia; *R. appendiculatus*: Tanzania, Zambia, Uganda and Kenya; *R. evertsi evertsi*: Tanzania, South Africa and Ethiopia; *R. pulchellus*: Ethiopia; *Rhipicephalus* ssp.: Ghana)

Acaricide resistance is generally less of a problem in multi-host than single-host ticks, and the development of acaricide resistance in several countries has been faster in *R. microplus* compared to multi-host ticks (Rodriguez-Vivas 2008; Rodriguez-Vivas et al. 2012a, 2014a, c). Since the first report of the development of resistance in *R. microplu*s populations to arsenicals in Australia in 1937, the progressive evolution of resistance in ticks affecting cattle to almost all of the available acaricides has frustrated the efforts of cattle producers to manage ticks and tick-borne diseases affecting their animals (Guerrero et al. 2014). Selected records of the geographic distribution of acaricide resistance in *R. microplu*s worldwide are listed in Table 4 and depicted in Fig. 2.

**Table 4:** Selected records of the geographic distribution of acaricide resistance in *R. microplus* worldwide

Continent/country	Reference	Acaricide or ML compound	Tick specie	Test
America				
USA	Miller et al. (2007b)	Permethrin	*R. microplus*	LPT
	Busch et al. (2014)	Coumaphos, permethrin, amitraz, ivermectin, fipronil	*R. microplus*	LPT
Mexico				
	Ortiz et al. (1995)	Dieldrin, lindane, coumaphos, diazinon, dioxathion, dimethoate, ethion, cypermethrin, deltamethrin, cypermethrin	*R. microplus*	LPT
	Fragoso et al. (1995)	Amitraz	*R. microplus*	LPT
	Soberanes et al. (2002)	Amitraz	*R. microplus*	LIT
	Li et al. (2004)	Carbaryl	*R. microplus*	LPT
	Rodriguez-Vivas et al. (2006a)	Diazinon, coumaphos, chlorfenvinphos	*R. microplus*	LPT
		Flumethrin, deltamethrin, cypermethrin	*R. microplus*	LIT
	Rodriguez-Vivas et al. (2006b)	Amitraz	*R. microplus*	LIT
	Rodriguez-Vivas et al. (2007)	Diazinon, coumaphos, chlorfenvinphos	*R. microplus*	LPT
		Flumethrin, deltamethrin, cypermethrin	*R. microplus*	LIT
	Rosado-Aguilar et al. (2008)	Amitraz	*R. microplus*	LIT
	Perez-Cogollo et al. (2010a)	Ivermectin	*R. microplus*	LIT
	Perez-Cogollo et al. 2010b)	Ivermectin	*R. microplus*	LIT
	Rodriguez-Vivas et al. (2011)	Cypermethrin	*R. microplus*	LPT
	Olivares-Pérez et al. (2011)	Amitraz, flumethrin, deltamethrin, cypermethrin, clorpyriphos, coumaphos, diazinon	*R. microplus*	LPT, LIT
	Fernandez-Salas et al. (2012c)	Cypermethrin	*R. microplus*	LPT
		Amitraz	*R. microplus*	LIT
	Fernandez-Salas et al. (2012b)	Diazinon, flumethrin, deltamethrin, cypermethrin	*R. microplus*	LPT
		Ivermectin	*R. microplus*	LIT
	Miller et al. (2013)	Fipronil	*R. microplus*	LPT
	Rodriguez-Vivas et al. (2013)	Ivermectin, amitraz	*R. microplus*	LIT
		Chlorpyrifos, coumaphos, cypermethrin, permethrin, fipronil	*R. microplus*	LPT
Argentina	Mangold et al. (2004)	Flumethrin	*R. microplus*	LPT
	Cutullé et al. (2013)	Amitraz, cypermetrin, flumethrin	*R. microplus*	AIT, LTT
	Lovis et al. (2013)	Amitraz, cypermethrin, flumethrin	*R. microplus*	LTT
	Cutullé et al. (2013)	Amitraz, deltamethrin	*R. microplus*	AIT, LTT
República Dominicana	Hagen et al. (1999)	Deltamethrin, flumethrin, cyfluthrin	*R. microplus*	LPT
Jamaica	Rawlins and Mansingh (1978)	Carbaryl, lindane, chlorfenvinphos	*R. microplus*	LIT
Cuba	Valdez et al. (1999)	Chlorfenvinphos	*R. microplus*	LPT
		Cyamizol	*R. microplus*	AIT
Venezuela	Coronado (1999)	Amitraz	*R. microplus*	AIT
Guatemala	Hagen et al. (1999)	Deltamethrin, flumethrin, cyfluthrin	*R. microplus*	LPT
Honduras	Hagen et al. (1999)	Deltamethrin, flumethrin, cyfluthrin	*R. microplus*	LPT
El Salvador	Hagen et al. (1999)	Flumethrin	*R. microplus*	LPT
Panama	Hagen et al. (1999)	Flumethrin	*R. microplus*	LPT
	Torrijos et al. (2015)	Cypermethrin	*R. microplus*	LPT
Costa Rica	Hagen et al. (1999)	Flumethrin	*R. microplus*	LPT
	Alvarez and Hernandez (2010)	Chlorpyrifos, coumaphos, flumethrin, deltamethrin, ivermectin	*R. microplus*	LPT
		Amitraz	*R. microplus*	LIT
Colombia	Benavides et al. (2000)	Cypermethrin, deltamethrin, coumaphos, clhorfenvinphos, diazinon, amitraz	*R. microplus*	LIT
		Amitraz	*R. microplus*	AIT
	Diaz and Vallejo (2013)	Cypermethrin	*R. microplus*	AIT
	Lopez-Arias et al. (2014)	Cypermetrhrin, amitraz	*R. microplus*	AIT
	Araque et al. (2014)	Amitraz, ethion	*R. microplus*	AIT
	Puerta et al. (2015)	Cypermethrin, amitraz	*R. microplus*	AIT
	Villar et al. (2016a)	Ivermectin	*R. microplus*	LIT
	Villar et al. (2016b)	Deltamethrin, amitraz, chlorpyrifos	*R. microplus*	AIT
Bolivia	Villarroel-Alvarez et al., 2006	Flumethrin, deltamethrin, cypermethrin	*R. micropus*	LPT
Uruguay	Castro-Janer et al. (2009)	Fipronil	*R. microplus*	LIT
	Castro-Janer et al. (2011)	Ivermectin	*R. microplus*	LIT
	Cuore and Solari (2014)	Ethion, cipermethrin, amitraz, fipronil, ivermectin	*R. microplus*	LPT, LIT
	Castro-Janer et al. (2015)	Fipronil	*R. microplus*	LIT
		Lindane	*R. microplus*	LPT
Brazil	Martins and Furlong (2001)	Doramectin, moxidectina	*R. microplus*	In vivo
	Li et al. (2004)	Amitraz	*R. microplus*	LPT
	Klafke et al. (2006)	Ivermectin	*R. microplus*	LIT
	Mendes et al. (2007)	Cypermethrin, deltamethrin, chlorpyriphos	*R. microplus*	LPT
	Castro-Janer et al. (2010)	Fipronil	*R. microplus*	LIT, LPT
	Klafke et al. (2010)	Ivermectin	*R. microplus*	LIT
	Klafke et al. (2011)	Ivermectin	*R. microplus*	LIT
	Andreotti et al. (2011)	Alpha-cypermethrin, cypermethrin, amitraz	*R. microplus*	AIT
	Mendes et al. (2011)	Deltamethrin, chlorpyriphos, cypermethrin	*R. microplus*	LPT
	Reck et al. (2014)	Chlorpyriphos, amitraz, cypermethrin, fipronil	*R. microplus*	LPT
		Ivermectin	*R. microplus*	LIT
		Fluazuron	*R. microplus*	AIT
	Klafke et al. (2016)	Amitraz	*R. microplus*	LPT
		Chlorpyriphos, cypermethrin	*R. microplus*	LPT
		Fipronil, ivermectin	*R. microplus*	LIT
		Chlorpyriphos, cypermethrin	*R. microplus*	AIT
Oceania				
New Caledonia	Brun et al. (1983)	Ethion	*R. microplus*	LPT
	Beugnet and Chardonnet (1995)	Fenvalerate, deltamethrin, flumethrin	*R. microplus*	LPT
	Bianchi et al. (2003)	Deltamethrin, ethion	*R. microplus*	LPT
	Ducornez et al. (2005)	Amitraz	*R. microplus*	LPT
Australia	Stone and Webber (1960)	BHC, DDT, dieldrin	*R. microplus*	LIT, AIT
	Stone and Meyers (1957)	Dieldrin	*R. microplus*	LIT, AIT
	Shaw (1966)	Carbophenothion, dioxathion, diazinon, parathion, carbaryl	*R. microplus*	LIT
	Nolan et al. (1989)	Cypermethrin, cyhalothrin	*R. microplus*	LIT, AIT
	Roulston et al. (1981)	Dimethoate, dioxathion, coumaphos, cyanophos, chlorpyrifos, dieldrin, DDT	*R. microplus*	
	Jonsson and Hope (2007)	Amitraz	*R. microplus*	LPT
	Lovis et al. (2013)	Flumethrin, cypermethrin, pyriprol	*R. microplus*	LTT
Asia				
India	Chaudhuri and Naithani (1964)	BHC	*R. microplus*	LIT, AIT
	Kumar et al. (2011)	Diazinon	*R. microplus*	ALT
	Sharma et al. (2012)	Deltamethrin, cypermethrin	*R. microplus*	LPT, AIT
	Shyma et al. (2013)	Deltamethrin, cypermethrin, diazinon	*R. microplus*	LIT, AIT
	Singh et al. (2014)	Cypermethrin	*R. microplus*	AIT
	Jyoti Singh et al. (2014)	Malathion	*R. microplus*	AIT
	Singh et al. (2015)	Amitraz	*R. microplus*	AIT
	Ghosh et al. (2015)	Deltamethrin, diazinon	*R. microplus*	AIT
	Shyma et al. (2015)	Deltamethrin, fipronil, flumethrin	*R. microplus*	AIT, LPT
	Gaur et al. (2016)	Deltamethrin, diazinon	*R. microplus*	LPT, AIT
Iran	Enayati et al. (2009)	Propetamphos	*R. bursa*	LPT
	Ziapour et al. (2016a)	Cypermethrin, lambda-cyhalothrin	*R. annulatus*	LPT
	Ziapour et al. (2016b)	Cypermethrin, lambda-cyhalothrin	*R. bursa*	LPT
Africa				
Ethiopia	Regassa and de Castro (1993)	Toxaphene	*R. decoloratus*	LPT
		Toxaphene	*R. evertsi evertsi*	LPT
	Yilma et al. (2001)	Dieldrine, diazinon, chlorfenvinphos, coumaphos	*B. decoloratus*	LPT
		Coumaphos	*R. evertsi evertsi*	LPT
	Feyera et al. (2015)	Diazinon	*R. pulchellus*	TIT
	Jobre et al. (2001)	Dieldrine, diazinon, clorfenvinphos, coumaphos	*B. decoloratus*	LPT
		Coumaphos	*R. evertsi evertsi*	LPT
Zimbabwe	Mazhowu (1995)	Dioxathion, flumethrin, cypermethrin, deltamethrin	*R. decoloratus*	LPT
Ghana	Kaljouw (2009)	Amitraz	*Rhipicephalus* ssp*.*	LPT
Tanzania	Kagaruki (1991)	Dieldrin, lindane	*R. microplus*, *R. decoloratus*, *R. evertsi evertsi*, *R. appendiculatus*	LPT
	Lourens and Tatchell (1979)	Toxaphene, BHC, dieldrin	*R. evertsi evertsi*	LPT
Benin	Adehan et al. (2016)	Alpha-cypermethrin, deltamethrin, amitraz	*R. microplus*	LPT
South Africa	Baker and Shaw (1965)	Toxaphene, lindane	*R. appendiculatus*	LIT, AIT, NIT
	Ntondini et al. (2008)	Amitraz, cypermethrin, chlorfenvinphos	*R. microplus*	LIT
		Chlorfenvinphos	*R. evertsi evertsi*	LIT
	Baron et al. (2015)	Amitraz	*R. microplus*	LPT
	Mekonnen et al. (2002)	Cypermethrin, chlorfenvinphos	*R. decoloratus*	LIT
	Mekonnen et al. (2003)	Chlorfenvinphos, cypermethrin	*R. decoloratus*	RET, ELT, LIT
		Amitraz	*R. decoloratus*	RET, ELT
		Chlorfenvinphos	*R. decoloratus*	LIT
		Permethrin	*R. decoloratus*	RET
	Lovis et al. (2013)	Pyriprol, cypermethrin, fenvalerate	*R. microplus*	LTT
	Coetzee et al. (1987)	Fenvalerate	*R. decoloratus*	LIT, AIT
Zambia	Luguru et al. (1987)	Dimethoate, dioxathion, chlorfenvinphos	*R. appendiculatus*	LPT
		Dieldrin, dimethoate, dioxathion, chlorfenvinphos	*R. decoloratus*	LPT
	Matthewson and Blackman (1980)	Dioxathion, toxaphene, chlorfenvinphos	*R. decoloratus*	LPT
	Muyobela et al. (2015)	Amitraz, cypermethrin	*R. microplus*, *R. appendiculatus*	LPT
Uganda	Vudriko et al. (2016)	Chlorfenvinphos, amitraz, cypermethrin, deltamethrin	*R. appendiculatus R. decoloratus*	LPT
Kenya	Baker and Shaw (1965)	Toxaphene, lindane	*R. appendiculatus*	LIT, AIT, NIT

*ML* macrocyclic lactone, *RET* reproductive estimate test, *ELT* egg-laying test, *TIT* ticks of equal size are immersed, *NIT* nymph immersion test, *R*. *Rhipicephalus*

## Strategies to minimize the development, progression and impact of resistance

The main strategies to delay the emergence of acaricide resistance include reduced frequency of application, modification of dose or concentration, use of mixtures, use of synergists, rotation between acaricide classes having differing mechanisms of action, preservation of untreated refugia and the application of biosecurity protocols to prevent introduction of resistant ticks (George et al. 2004). To reduce the development of resistance, the knowledge of the tick species present and the resistance status should be considered before the selection of acaricides. Cases of field resistance should be confirmed in the laboratory.

### Reducing frequency of application

Any effective non-acaricidal control agent that can be applied to control ticks should reduce the requirement for acaricide use and therefore reduce selection pressure on acaricides. Commonly used or discussed control methods include manual removal, selection of cattle with high resistance to infestation, use of plants and plant extracts, vaccination and biological control agents (Rodriguez-Vivas et al. 2014b). These approaches are all discussed in detail below.

### Synergized pesticides and pesticide mixture formulations

Synergism between different groups of ectoparasiticides has been used in several countries to control insects and ticks for many years (Li et al. 2007; Barré et al. 2008; Rodriguez-Vivas et al. 2013). Knowles (1982) demonstrated that amitraz and chlordimeform can act as synergists of OC, OP, carbamate and SP insecticides. Subsequent publications confirmed the synergism of amitraz and pyrethroids against insects and ticks (Usmani and Knowles 2001; Li et al. 2007), amitraz and fipronil against ticks (Prullage et al. 2011) and pyrethroids and neonicotinoids against mosquitoes (Ahmed and Matsumura 2012). Under laboratory conditions, Li et al. (2007) showed that adding amitraz to permethrin led to a strong increase in larval mortality of a highly pyrethroid-resistant strain of *R. microplus.* The synergism between deltamethrin and amitraz was subsequently confirmed in a field trial on a farm in New Caledonia (Barré et al. 2008).

The main synergists that have been used as ixodicide action potentiators for tick control are piperonyl butoxide (PBO) (a cytochrome P450 monooxygenase inhibitor), triphenylphosphate (TPP, an esterase inhibitor), diethyl maleate (DEM, an inhibitor of glutathione-S-transferases) and verbutin (an inhibitor of certain cytochrome P450 isoforms) (Li et al. 2007). Metabolic enzyme defense systems including the cytochrome P450 monooxygenases and esterases are present at a ‘baseline level’ in arthropods. In resistant arthropods, their activity can be elevated to detoxify pesticides (Young et al. 2006). Li et al. (2010) demonstrated that the use of a reduced PBO and verbutin concentrations potentiates the action of permethrin, coumaphos and amitraz. The verbutin demonstrated greater synergism than PBO to control *R. microplus* larvae resistant to coumaphos (synergism index (SI) = 1.5–6.0 vs. 0.9–1.6) and amitraz (SI = 1.8–1.5 vs. 0.9–2.5), but similar synergism for permethrin (SI = 2.1–4.4 vs. 2.1–3.6). Rodriguez-Vivas et al. (2013) evaluated the efficacy of cypermethrin, amitraz and PBO mixtures, through in vitro laboratory bioassays and in vivo on-animal efficacy trials, for the control of resistant *R. microplus* on cattle in the Mexican tropics. The authors showed that the mixture of cypermethrin + amitraz + PBO was most effective for killing resistant tick in vitro and in vivo conditions.

### Rotation of acaricides

Rotation refers to the alternation of the use over time of two or more active ingredients with differing modes of action and no potential for cross-resistance (Rodriguez-Vivas et al. 2014b). Thullner et al. (2007) evaluated an acaricide rotation strategy for managing resistance in *R. microplus* under laboratory and field conditions in Costa Rica. The strain that they studied exhibited resistance to deltamethrin and a very low resistance to Ops, and it was kept under selection pressure for 9 to 11 generations by using deltamethrin or coumaphos, either exclusively or in rotation. In the sub-strains selected continuously with coumaphos or coumaphos and deltamethrin in rotation, no significant increase in resistance to deltamethrin was observed. In Australia, Jonsson et al. (2010b) treated calves with *R. microplus* amitraz resistance, with amitraz alone, spinosad alone or a rotation between spinosad and amitraz every 2 months over 4 years. The treatments with spinosad and spinosad in rotation with amitraz treatments resulted in the loss of amitraz resistance and a return to full or almost full susceptibility to amitraz. The loss of resistance to amitraz suggested that rotation of amitraz with other acaricides might prolong the useful life of the product.

Besides these laboratory and field studies to demonstrate that rotations show some promise for the management of acaricide resistance, the results can be expected to vary depending on the fitness and mode of inheritance of a particular form of resistance (George et al. 2004). Amitraz is an example of an acaricide that might possibly be used effectively in a rotation program because there is some evidence of loss of resistance to amitraz in populations of ticks on farms where cattle were treated with other alternatives and the mode of inheritance appears to be recessive. Conversely, in *R. microplus* resistant to SP and OP, reversion to susceptibility is difficult because it has been demonstrated that resistance persist to OP and SP for several years (Rodriguez-Vivas et al. 2005, 2011). Additionally, Aguilar-Tipacamu et al. (2008) demonstrated that the main mode of inheritance of SPs in *R. microplus* is by a partially dominant trait.

### Correct application of acaricide and macrocyclic lactones

Short time intervals between successive acaridide treatments are associated with an increase in the proportion of a population that is resistant to an acaricide. In New Caledonia, Bianchi et al. (2003) reported that farmers are accustomed to controlling ticks every month or whenever they observe a substantial tick infestation. When the ticks become resistant, the first reaction of the farmers is to decrease the interval between treatments. Frequent applications of acaricides and its association with acaricide (Sutherst 1979; Jonsson et al. 2000; Rodriguez-Vivas et al. 2006a) and ML resistance in *R. microplus* (Fernandez-Salas et al. 2012a) have been demonstrated worldwide.

In countries with well-developed systems of agricultural pesticide regulation, there is a little chance that the manipulation of acaricide concentration will ever be an option as a method to delay the emergence of resistance, because legislation generally prescribes their use only at the acaricide concentrations specified on their label (Guerrero et al. 2014). However, in some developing countries, acaricide concentrations are manipulated by farmers from time to time (Higa et al. 2016).

Dosage determination of injectable formulations of ML to control ticks and nematodes on cattle is based on the body weight of individual animal. However, on cattle ranches with low income, cattle farmers calculate the weight of animals by visual appraisal. This practice could obviously enable misuse of drugs which would possibly lead to treatment failures as a result of inappropriate dosing by underestimation of the live weight. Despite this well-known statement in relation to nematodes, visual estimation of body weight to treat cattle with ivermectin has not been associated with ivermectin resistance in *R. microplus* (Fernandez-Salas et al., 2012a). Further studies are needed to verify whether variation in dose of ML has any effect on the frequency of resistant alleles under laboratory and field conditions.

The method of acaricide application is significantly related to tick resistance. The hand spray does not sufficient wet cattle, and this can be induced by insufficient pump pressure or the obstruction of nozzles. Bianchi et al. (2003) mentioned that this defect could select resistant strains; however, Jonsson et al. (2000) found in Australia that the use of a spray race to apply acaricides was associated with higher probabilities of Lamington (resistant to flumethrin) and Parkhurst resistance (resistant to all synthetic pyrethroids), while the use of a hand spray reduced the likelihood of Ulam resistance (resistant to amitraz). The hand spray method leaves many ticks completely unexposed to acaricides, and the relative fitness of susceptible homozygotes would be increased, delaying the development of resistance. Further studies are needed to clarify this statement.

## Non-acaricidal control of ticks

### Manual removal

The manual removal of ticks is mainly practised in developing countries and is only able to be applied on small farms where the number of tick-infested cattle is low. Muhammad et al. (2008) noted that care is required when removing ticks from animals because ticks can also transmit deadly pathogens to humans (i.e. Crimean–Congo hemorrhagic fever virus, usually associated with ticks of the genus *Hyalomma*). WingChing-Jones (2015) studied the impact of manual removal of *R. microplus* ticks on tick densities on Jersey dairy cows over 4 years in Costa Rica. During the morning milking, twice a week, ticks with a size between 5 and 10 mm long were counted and removed. The technique reduced the tick population by 21%; however, its efficacy was conditional on the number of animals in the herd and personnel availability.

### Host resistance

Host resistance of cattle to ticks is associated with a reduced number of ticks feeding to engorgement, reduced egg production and reduced egg viability (Wikel 1996). Differences in the ability of cattle to become resistant to ticks, whether *Bos indicus* or *Bos taurus* or within the *B. taurus* breed, have long been recognized, as has the fact that the ability to acquire resistance is heritable (Utech et al. 1978). In *Bos indicus*-cross cattle for example, heritability estimate for burden of *R. microplus* is moderate (*h* ^2^ = 0.34, Mackinnon et al. 1991). It has also been shown that *Bos indicus* or their crossbreeds are more able to survive babesiosis (a tick-borne disease transmitted by *B. bovis* and *B. bigemina*) than *B. taurus* animals (Bock et al. 1997). The mechanisms of resistance to infestation with ticks have been reviewed elsewhere (Jonsson et al. 2014). The potential reduction in acaricide requirement arising from concerted selection and breeding of cattle for increased host resistance is very substantial. Indicine cattle carry between 10 and 20% of the number of ticks that taurine cattle would carry given the same level of larval tick challenge (Jonsson et al. 2014). Whereas the most rapid gains in host resistance can be made by replacing taurine cattle with indicine breeds or crossbreeds, molecular genetic markers of host resistance have been identified and with further development hold promise of more rapid selection for host resistance within breeds (Porto Neto et al. 2011).

### Release of sterile male hybrids

It has been shown that *R. annulatus* × *R*. *microplus* matings produce fertile females and sterile males (Osburn and Knipling, 1982). Backcrossing of the fertile female progeny also produces sterile males and fertile females through three to six generations. To be successful, release of hybrid ticks must be into small populations, for example where there is a new outbreak or where there is already a high degree of control by other means (Hillburn et al. 1991). Problems with this method of control include the cost of production of hybrids, the effects of moderate infestations of hybrids over the period of eradication and the risk of an extended range of hybrid or *R. annulatus* ticks (Jonsson 1997).

### Enviromental and animal management

Management of refugia (parasitic populations that have not been exposed to a particular drug and hence still contains a large proportion of susceptible organisms) by pasture rotation and strategic administration of anthelmintics, treating only the most heavily parasitized animals, has been used in horses and ruminants to delay progression of helminth resistance (Rodriguez-Vivas et al. 2014b). This type of management can be applied for tick control. The following is a brief overview of the major enviromental and animal management practices that contribute to control ticks.

#### Plant species that are unfavourable to ticks

Some plants have been shown to act as attractants for ticks; Wilson et al. (1989) demonstrated that *Stylosanthes scabra* (a tropical legume) can trap between 12 and 27% larvae in the sticky exudate of glandular trichomes on stems and leaves. However, the effectiveness for tick control is limited by the proportion of this plant in pastures and the physiological state of the plant. Additionally, the African shrub *Acalypha fruticosa* is reported to attract larvae of *R. appendiculatus*, which lie quiescent on the underside of the leaf plant (Hassan et al. 1994).

#### Grazing management

Pasture management in which grazing patterns are used to interrupt the life cycle of ticks can be use in an integrated tick control (Stachurski and Adakal 2010; Abbas et al. 2014). Pasture spelling was implemented to starve larval ticks by rotating cattle into ‘clean’ paddocks at specified intervals. In Australia, spelling periods of 3–4 months were considered necessary, but such long periods sometimes have adverse effects on pasture quality. Pasture spelling was used effectively in certain situations but had limited appeal to producers because of managerial difficulties, the cost of fencing and pasture irrigation facilities and the possible adverse effect on pasture quality (Elder et al. 1982).

#### Pasture burning

Burning pasture is a widely used practice for controlling ticks in many countries like South Africa, Zambia, Australia, the USA and Mexico (Abbas et al. 2014). Fire directly affects ticks due to the exposure of larvae, adult females and eggs to high temperatures. Indirectly, it has an effect by the destruction of the vegetation layer that serves as protection to the ticks (Rodriguez-Vivas et al. 2014b).

#### Animal nutrition

Energy and protein are important in mediating acquired resistance to ticks (Wikel 2013). In a field study carried out in eastern Queensland, Australia, feeding on poor-quality pastures resulted in a significant loss of resistance in the *Bos taurus* and *B. indicus* × *B. taurus* steers and heifers to *R. microplus*. Sutherst et al. (1983) mentioned that animals grazing native pastures, with poor-quality feed in late-autumn and winter, suffered substantial losses of resistance of ticks.

#### Plant extracts and essential oils to control ticks

Many species of plants have been evaluated for acaricidal activity, with the species studied mainly being members of the families Poaceae, Fabaceae, Lamiaceae, Verbenaceae, Piperaceae and Asteraceae (Borges et al. 2011; Muyobela et al. 2016; Dantas et al. 2016). Some studies have identified secondary metabolites (terpenes, stilbenes, coumarins, alcohols, acids, sulfurated compounds and aldehydes) of essential oils and plant extracts, associated with acaricidal effects against the genera *Amblyomma*, *Rhipicephalus*, *Hyalomma*, *Dermacentor*, *Argas* and *Ixodes* (Pamo et al. 2005; Cetin et al. 2010). In this section, only few examples of plant extracts and essential oils with acaricide property will be described. For more comprehensive article, we recommend Borges et al. (2011) and Rosado-Aguilar et al. (2017).

Srivastava et al. (2008) evaluated the ethanolic crude extract of *Azadirachta indica*, *Mangifera indica*, *Prunus persica*, *Curcuma longa* and *Psidium guajava*. *Azadirachta indica* seed extract was more effective (80%) than *P. persica* seed (70%) and *A. indica* leaf (30%). The extracts prepared from *A. indica* bark, *P. persica* leaf and *M. indica* bark had no effect on the adults of *R. microplus*, while only 10% of adults died when treated with the extract of *C. longa*. Fernandez-Salas et al. (2011) evaluated the acaricidal activity of acetone–water extracts from the fresh leaves from four tannin-rich plants (*Acacia pennatula*, *Leucaena leucocephala*, *Piscidia piscipula* and *Lysiloma latisiliquum*) against the larvae and adult ticks of *R. microplus*. The following mortality rates were obtained: 54.8% for *A. pennatula*, 66.7% for *L. leucocephala*, 88.1% for *P. piscipula* and 56.0% for *L. latisiliquum*. However, none of the evaluated plants showed acaricidal activity against adult ticks. Sardá-Ribeiro et al. (2008) evaluated the hexane extract from the aerial parts of *Calea serrata* to control larvae and adults of *R. sanguineus* and *R. microplus*, showing 100% mortality in the larvae of both tick species and a reduction in oviposition of 11–14%. In two studies conducted by Broglio-Micheletti et al. (2009, 2010), extracts and commercial products using *A. indica* were evaluated. Ethanolic extracts from leaves and hexanic extracts from seeds had efficacy of 2.3 and 38.4, respectively, on *R. microplus* female reproduction (Broglio-Micheletti et al. 2009). Efficiency of commercial formulations of alcoholic and hexanic extracts from seeds was from 17 to 73% (Broglio-Micheletti et al. 2010). In another study, the essential oil of *Cymbopogon winterianus* was avaluated against larvae and engorged females of *R. microplus*. Total inhibition of eclosion was observed at a concentration of 7.1 and 100% of larval mortality at concentrations between 5.5 and 7.1%. The principal components of the essential oil, i.e. geraniol, citronellal and citronellol, were tested against engorged females, and the best results were observed for geraniol and citronella. Rosado-Aguilar et al. (2010) studied the acaricidal activity of crude extracts and fractions from stems and leaves of *Petiveria alliacea* against larvae and engorged females of *R. microplus.* Methanolic extracts of leaves and stems of *P. alliacea* showed 100% mortality of larvae. The methanolic extracts of stem and leaves on engorged females showed 86 and 26% of mortality, respectively, egg laying inhibition of 91 and 40%, respectively, and hatchability inhibition of 17 and 26%, respectively. Purification of the active stem methanolic extract showed six main compounds: benzyl disulfide, benzyl trisulphide, *cis*-stilbene, methyl esters of hexadecanoic acid, octadecadienoic acid and octadecenoic acid. To validate the acaricidal activity of these compounds, Arceo-Medina et al. (2016) evaluated the six commercially available compounds individually and in 57 combinations. The mixtures based on the benzyl trisulphide + benzyl disulfide pairing produced a synergistic effect against acaricide-resistant *R. microplus* larvae and engorged females and were therefore the most promising combination for controlling this ectoparasite. Recently, Avinash et al. (2017) studied the in vitro acaricidal activity of neem-coated silver nanoparticles on deltamethrin resistance *R. microplus*. These nanoparticles produced 93% mortality at 50 ppm and efficacious inhibition of oviposition and reproductive index of engorged females.

Although several plant extracts have been tested against *R. microplus* in laboratory conditions, only a few of them have also been evaluated on *R. microplus*-infested animals in order to validate the results obtained (Borges et al. 2011). One expected advantage from the use of any effective botanical compounds would be slow development of resistance because there is usually a mixture of different active componds with different mechanisms of action.

#### Vaccination

Immunization against ticks at present seems appealing due to its potential for the prevention of drug-resistant ticks and reduction of environmental damage (Guerrero et al. 2012). Tick antigens are usually classified as either exposed or concealed antigens. Exposed antigens are those that naturally come into contact with the host immune system during tick feeding (i.e. antigens from the salivary gland and its secretions and cuticle), and animals are continually exposed to this class of antigen during infestation. Conversely, concealed antigens (including some antigens from gut epithelium) are not exposed to the host immune system during tick feeding, and therefore, repeated vaccinations are required to maintain high antibody titers (Manjunathachar et al. 2014).

Willadsen et al. (1989) first identified the Bm86 antigen-concealed antigen from the midgut of engorged female *R. microplus* tick and demonstrated its efficacy as a vaccine in both its native and recombinant forms. The authors subsequently developed an expression system for Bm86, and it was commercialized in Australia as TickGARD® (Willadsen et al. 1995). Bm86-based vaccines cause leakage of gut content into the haemocoele of ticks, slightly reducing the number of females engorging, their mean weight and fecundity and reducing larval production. Another commercial vaccine containing a recombinant Bm86 antigen (Gavac®) was released in Mexico, Argentina and Colombia in 1997 (Canales et al. 1997). Controlled pen and field trials in Mexico provided evidence of the effect of recombinant Bm86 vaccination for the control of *R. microplus* and *R. annulatus* infestations (de la Fuente et al. 2007).

The mechanism of Bm86-based vaccine against tick infestation is based on polyclonal antibody response against the concealed antigen. Regional variation in the sequence of Bm86 has been proposed to influence the efficacy of Bm86-based recombinant vaccines (Manjunathachar et al. 2014). Studies in Argentina revealed polymorphisms in the Bm86 gene that affected expression of the gene and resulted in the production of a soluble rather than a membrane-bound protein in ticks that were apparently resistant to vaccination with the original Bm86 (Garcia-Garcia et al. 2000). Field trials of the TickGARD® vaccine in some areas of Brazil showed low levels of efficacy (Pereira et al. 2008). Gavac® remains commercially available in some Latin American countries, but TickGARD® is no longer commercially available in Australia (Schetters et al. 2016).

Research towards the development of more effective vaccines has received considerable support in recent years, and there are many promising candidates as well as studies to improve the efficacy and delivery of the existing antigen. A detailed overview and evaluation of all publicly reported candidates is beyond the scope of this review, and the subject is covered elsewhere (e.g. Schetters et al. 2016).

#### Biological control

Biological control is defined broadly as the use of live organisms to reduce the populations of pest/pathogenic organisms. A distinction is often made between biopesticides and biological control agents. Biopesticides are live organisms or products thereof, which must be applied directly and whenever needed to the pest to control it. Biopesticides do not survive, establish populations and proliferate in the environment and are therefore not expected to have a persistent effect arising from their survival. In contrast, biological control agents are expected to establish in the environment and to have an ongoing effect on the pest species. They can be considered as depressing the equilibrium population of the pest in their environment. Examples of biological control agents include predators, pathogens, parasites and resistant plants. Research has been conducted on nematodes (*Heterorhabditis* spp. and *Steinernema* spp.), ants (*Solenopsis germinata*, *S. saevissima* and *Ectatomma cuadridens*) and many bird species (Samish et al. 2004; Ojeda-Chi et al. 2011). Entomopathogenic fungi and *Bacillus thuringiensis* and its products are generally considered to be biopesticides. General predators can sometimes affect the size of a tick population in nature, but manipulating their populations to reduce tick numbers would require large increases in the predator population, which could also cause large changes in populations of non-target species in natural areas (Samish et al. 2004).

The entomopathogenic fungi that have been evaluated for the control of *R. microplus* are mainly *Beauveria bassiana*, *Lecanicillium lecanii* and *Metarhizium anisopliae*, which have shown potential efficacy in the control of various tick developmental stages (egg, larva, nymph, adult) (Ojeda-Chi et al. 2011). Laboratory and field evaluations of *M. anisopliae* for the control of *R. microplus* have been documented worldwide (Samish et al. 2004). Frazzon et al. (2000) studied 12 strains of *M. anisopliae* and found four strains that killed 50% of engorged females after a single fungal immersion. During a subsequent immersion (1 × 10^7^ conidia/ml), nine strains killed 100% of ticks. Fernandez et al. (2005) found a highly effective *M. anisopliae* strain that killed 100% of engorged females, both resistant and susceptible to acaricides, with a 1 × 10^8^ conidia/ml concentration. Gindin et al. (2001) also found a *M. anisopliae* strain that killed 80–100% engorged females of *R. annulatus*. In the Mexican tropics, Ojeda-Chi et al. (2010) tested the effect of two strains of *M. anisopliae* to control *R. microplus* under laboratory and field conditions (larvae on vegetation). The efficacies in laboratory conditions at 1 × 10^8^ conidia/ml concentration for larvae and adult stages were 45–100 and 100%, respectively. The efficacy of *M. anisopliae* to control *R. microplus* larvae on vegetation varied from 68 to 100%. General efficacy of *M. anisopliae* to control *R. microplus* in in vitro and in vivo (on animals and on vegetation) conditions are 50–100 and 36–90%, respectively (Ojeda-Chi et al. 2011). The efficacy of *M. anisopliae* varies depending on the strain and conidial concentration (Fernandes et al. 2004; Samish et al. 2004). Kirkland et al. (2004) mentioned that virulence depends on the ability of *M. anisopliae* to penetrate directly through the tick cuticle using enzymatic and physical mechanisms. Despite the promising laboratory results with fungal biopesticides for the control of ticks, in vivo studies have not repeatably yielded promising results.

## Integrated tick management

Integrated tick management (ITM) consists of the systematic combination of two or more technologies to control pest populations which adversely affect the host species, while maintaining adequate levels of animal production. The aim of this management is “to achieve pest control in a more sustainable, environmentally compatible and cost-effective manner than is achievable with a single, stand alone technology” (Willadsen 2006). In the development of approaches which allow effective management of tick populations, which minimize non-target effects and preserve the availability of the existing acaricides, it is essential to develop more fully the use of ITM. In such approaches, combinations of management tools may be deployed as and when necessary, with acaricide available as just one component, to be used in appropriate circumstances (Guerrero et al. 2014). A wide range of new tools are becoming available to assist in this goal. These include molecular techniques, which can provide powerful new insights into diagnosis, spatial distribution of ticks, acaricide resistance of ticks, simulation modelling, satellite imagery, anti-tick vaccines and biological control (Jonsson 2004; Estrada-Peña and Venzal 2006; Alonso-Díaz et al. 2007; de la Fuente et al. 2007; Jonsson and Hope 2007; Rodriguez-Vivas et al. 2007; Ojeda-Chi et al. 2010). However, there is little evidence that these tools are being applied to any extent in the field.

In Mexico, the anti-tick vaccine (Gavac®) and acaricide treatments have been used together to control *R. microplus* ticks. Redondo et al. (1999), using an integrated system employing vaccination with amitraz treatments and Gavac®, under field conditions achieved nearly 100% control of *R. microplus* populations resistant to OPs and SPs. This method effectively controls tick infestations while reducing the number of chemical acaricide treatments and consequently the rise of *R. microplus* populations resistant to acaricides. Furthermore, in a farm using this ITM for 10 years, a substantial reduction of acaricide treatments was achieved (from 24 to 7–8 per year) with consequent reduction in tick infestation from 100 to 20 adult ticks per animal (de la Fuente et al. 2007).

Bahiense et al. (2006) evaluated the combined use of the entomopathogenic fungus *M. anisopliae* and deltamethrin against *R. microplu*s larvae that were resistant to SP. High mortality rates were observed when deltamethrin was associated with the entomopathogen. The potential utilization of associated chemical acaricides with biological agents could stimulate the use and consolidation of biological control for animal parasites among farmers and practitioners (Webster et al. 2015).

The use of tick-resistant cattle breeds (*B. indicus* and their crosses), host management (i.e. lowering the stocking rate), selection application of acaricide during annual season when they will be most effective and pasture rotation and spelling can be useful components of an ITM (Rodriguez-Vivas et al. 2014b).

There are many studies demonstrating that the integrated management of parasites is the best option to increase the productive capacity of animals; however, studies are mainly based on the control of one type of parasite (i.e. ticks) by the use of several control approaches. Because internal (i.e. gastrointestinal nematodes) and external parasites (i.e. ticks, flies, lice) of cattle occur in natural conditions simultaneously, it is necessary to control different types of parasites. The main challenge that exists worldwide is the efficient use of an integrated program of parasites in livestock (unless it controls ticks, gastrointestinal nematodes and hematophagous flies) through the implementation of coordinated strategies of chemical and non-chemical control (Rodriguez-Vivas et al. 2014b).

## Conclusions

The control of *Rhipicephalus* ticks, especially *R. microplus*, is achieved mainly by chemical acaricides and ML. However, there is measureable resistance to most of the compounds that are commercially available, and this can be expected to increase. There is a need to develop and validate the efficacy of strategies for tick control that will delay the emergence of resistance. Selection pressure can be reduced by including non-acaricide-based controls (i.e. integrated tick management) and by using targeted treatments to maximize refugia. Mixtures of compounds will increasingly be required in response to increased prevalence of acaricide resistance. Biosecurity should be given high priority to reduce the dispersal of resistance-conferring variants. The value of rotation of acaricides should be investigated for a range of compounds under field conditions.
